# Plasma Levels of Soluble Urokinase-Type Plasminogen Activator Receptor Associate with the Clinical Severity of Acute Puumala Hantavirus Infection

**DOI:** 10.1371/journal.pone.0071335

**Published:** 2013-08-21

**Authors:** Tuula K. Outinen, Laura Tervo, Satu Mäkelä, Reetta Huttunen, Niina Mäenpää, Heini Huhtala, Antti Vaheri, Jukka Mustonen, Janne Aittoniemi

**Affiliations:** 1 Department of Internal Medicine, Tampere University Hospital, Tampere, Finland; 2 School of Medicine, University of Tampere, Tampere, Finland; 3 Department of Clinical Microbiology, Fimlab Laboratories, Tampere, Finland; 4 School of Health Sciences, University of Tampere, Tampere, Finland; 5 Department of Virology, Haartman Institute, University of Helsinki, Helsinki, Finland; Karolinska Institutet, Sweden

## Abstract

**Objectives:**

Urokinase-type plasminogen activator receptor is a multifunctional glycoprotein, the expression of which is increased during inflammation. It is known to bind to β_3_-integrins, which are elementary for the cellular entry of hantaviruses. Plasma soluble form of the receptor (suPAR) levels were evaluated as a predictor of severe Puumala hantavirus (PUUV) infection and as a possible factor involved in the pathogenesis of the disease.

**Design:**

A single-centre prospective cohort study.

**Subjects and Methods:**

Plasma suPAR levels were measured twice during the acute phase and once during the convalescence in 97 patients with serologically confirmed acute PUUV infection using a commercial enzyme-linked immunosorbent assay (ELISA).

**Results:**

The plasma suPAR levels were significantly higher during the acute phase compared to the control values after the hospitalization (median 8.7 ng/ml, range 4.0–18.2 ng/ml *vs.* median 4.7 ng/ml, range 2.4–12.2 ng/ml, *P*<0.001). The maximum suPAR levels correlated with several variables reflecting the severity of the disease. There was a positive correlation with maximum leukocyte count (r = 0.475, p<0.001), maximum plasma creatinine concentration (r = 0.378, p<0.001), change in weight during the hospitalization (r = 0.406, p<0.001) and the length of hospitalization (r = 0.325, p = 0.001), and an inverse correlation with minimum platelet count (r = −0.325, p = 0.001) and minimum hematocrit (r = −0.369, p<0.001).

**Conclusion:**

Plasma suPAR values are markedly increased during acute PUUV infection and associate with the severity of the disease. The overexpression of suPAR possibly activates β_3_-integrin in PUUV infection, and thus might be involved in the pathogenesis of the disease.

## Introduction

Hantaviruses cause two clinical syndromes in humans, hemorrhagic fever with renal syndrome (HFRS) in Eurasia and hantavirus cardiopulmonary syndrome (HCPS) in the Americas [1], [2]. Puumala hantavirus (PUUV), carried by the bank vole, causes a mild HFRS called nephropathia epidemica (NE) [1], [2]. NE is prevalent in Finland, elsewhere in Scandinavia, European Russia, and many parts of Central-Western Europe [1], [2]. In Europe, PUUV causes most HFRS cases, and in Finland, 1,000–3,000 serological PUUV infection diagnoses are made annually (http://www3.ktl.fi) [3].

The clinical severity of NE varies from subclinical disease to fatal cases. However, the mortality rate is low, about 0.1% [4]. Typical symptoms are sudden high fever, headache, abdominal pain, nausea, backache, and visual disturbances, while serious hemorrhagic manifestations are uncommon [5]–[8]. Signs of renal involvement are proteinuria, hematuria, and oliguria, the latter being followed by polyuria [5]–[8]. During the oliguric phase, about 5% of hospitalized patients need transiently hemodialysis treatment [6]–[8]. Usual laboratory findings are leukocytosis, thrombocytopenia, anemia, elevation of plasma C-reactive protein (CRP) and creatinine levels, as well as proteinuria and hematuria [6], [7]. Radiological pulmonary manifestations have been detected in about one-third of hospitalized patients [9], [10].

The pathogenesis of NE is incompletely understood. An important feature in hantavirus infections is universally increased capillary permeability leading to vascular leakage, but the mechanisms behind this phenomenon are unclear [11]–[13]. The endothelium of the small vessels in various organs is the primary target of hantavirus infection and β_3_-integrins mediate the cellular entry of pathogenic hantaviruses [14], [15]. It has been suggested that immunological factors rather than direct cytotoxicity are essential in the pathogenesis, since no obvious damage to the endothelial cells is seen [11]–[13].

Several biomarkers have been shown to serve as indicators of the severity of PUUV infection. The plasma levels of interleukin (IL)-6, pentraxin-3 (PTX3), and indoleamine 2,3 dioxygenase (IDO), all elements of the innate immunity, as well as of cellular damage -reflecting cell-free DNA (cf-DNA) predict the outcome of PUUV infection [16]–[19]. So does the urine levels of the transcription factor necessary for the generation of type 2 T-cells, GATA-3 [20]. On the contrary, although plasma CRP is elevated in almost all of the patients with PUUV infection, high CRP level does not indicate a more severe disease [19].

Urokinase-type plasminogen activator receptor (uPAR) is a multifunctional glycoprotein, the expression of which is increased during infection and inflammation [21], [22]. It is expressed on several different cell types, including monocytes, activated T-lymphocytes, macrophages, neutrophils, endothelial cells, and kidney podocytes [21], [22]. uPAR interacts with several molecules mediating immune system signals and promotes the migration and adhesion of leukocytes by binding to β-integrins [21], [22]. Cell-cell contact has been shown to enhance the release of the soluble form of the receptor (suPAR) by endothelial cells, indicating a regulatory role of suPAR in cell adhesion [23]. The levels of plasma suPAR are considered to represent the degree of immunoactivation [22]. Plasma suPAR has been found to be increased as well as predict disease severity in various conditions, e.g. autoimmune diseases, cancer, malaria, tuberculosis, sepsis, and human immunodeficiency virus (HIV) infection [22], [24]–[30].

In the present study, our aim was to evaluate the association of suPAR with the severity of acute PUUV infection and also assess the possible role of suPAR in the pathogenesis of the disease.

## Materials and Methods

### Ethics statement

All subjects gave a written informed consent before participation and the study was conducted according to the principles expressed in the Declaration of Helsinki and approved by the Ethics Committee of Tampere University Hospital.

### Patients

The study cohort consisted of 97 prospectively collected adult patients with acute NE. The diagnosis of acute PUUV infection was serologically confirmed in all cases [31]. The patients were treated at the Tampere University Hospital (Tampere, Finland) from November 2001 to February 2009. The median patient age was 41 (range 22–77) years, and 63 (65%) were males. Part of the patients (10–83 patients of the present cohort depending on the previous study) has participated also in our previous studies [16]–[20], [32]–[35]. All the patients treated during November 2001 and February 2009 were included in the study, if there was plasma left for the analyses of suPAR concentration.

### Study protocol

All 97 patients were examined during the acute phase of NE. A detailed past and current medical history was obtained, and a careful physical examination was performed. Blood samples were collected between 7:30–9:30 in the morning for two consecutive weekdays after hospitalization for the analysis of plasma suPAR. Blood samples for the determination of plasma cf-DNA, PTX3, IL-6, CRP, creatinine, and serum kynurenine (Kyn) and tryptophan (Trp) levels, as well as the blood cell counts were collected for up to five consecutive days. Other blood samples were taken according to the clinical needs of the patient.

The highest and the lowest values of the various variables measured during hospitalization for each patient were designated as the maximum and minimum values.

Eighty-four (87%) of the 97 patients were also studied at the out-patient clinic 1–4 weeks after the hospital period. The plasma samples taken at the out-patient clinic after the hospital treatment were regarded as control samples.

### Methods

EDTA-treated plasma samples for suPAR determination were collected from patients during hospitalization and at the out-patient clinic and stored at −70°C until required for analysis. Plasma suPAR levels were determined using a commercial enzyme-linked immunosorbent assay (ELISA) (suPARnostic® Standard kit;ViroGates A/S, Birkerød, Denmark) according to the manufacturer's instructions.

Plasma CRP and creatinine levels were analyzed using Cobas Integra analyzer (F. Hoffman-La Roche Ltd, Basel, Switzerland). Blood cell count was completed by hematological cell counters by Bayer. Plasma IL-6 concentrations were determined as previously described [36]. IDO level can be measured by determining the ratio of Kyn to Trp in serum [37] by reverse-phase high performance liquid chromatography (HPLC) as previously described [38]. The Kyn/Trp ratio was calculated by relating concentrations of Kyn to Trp. Plasma PTX3 determinations were performed by using a commercially available human pentraxin-3 immunoassay (Quantikine, R&D Systems, Inc., Minneapolis, MN), following the manufacturer's instructions. Plasma cf-DNA levels were determined as previously described [16]. Plasma IL-6, PTX3, and cf-DNA, as well as serum Kyn and Trp concentrations were measured afterwards from frozen samples.

### Statistical Analyses

In order to describe the data, medians and ranges were given for continuous variables and numbers and percentages for categorical variables. Groups were compared using the Mann-Whitney *U*- test. Correlations were calculated by the Spearman's rank correlation test. Wilcoxon's test was used to compare two related samples. All tests were two-sided, and statistically significant *P*-values are given. All analyses were made with the SPSS (version 20) statistical software package (IBM, Chicago, IL).

## Results

The clinical characteristics of the patients are shown in Table 1 and the laboratory variables measured during hospitalization in Table 2. None of the patients was in clinical shock at the time of admission and five patients (5%) needed hemodialysis treatment during the hospital stay. No deaths occurred.

**Table 1:** Clinical data of 97 patients with acute Puumala hantavirus infection.

	Median	Range
Age (years)	41	22–77
Duration of fever before hospital admission (days)	4	1–15
Total duration of fever (days)	7	2–19
Length of hospital stay (days)	6	2–15
Change in weight during hospitalization (kg)	2.1	0–12.0
Minimum urinary output (ml/day)	1400	50–5800

**Table 2:** Laboratory data of 97 patients with acute Puumala hantavirus infection.

	Median	Range
suPAR max (ng/ml)	8.7	4.0–18.2
Creatinine max (µmol/l)	175	51–1499
Platelets min (10^9^/l)	61	9–187
Hematocrit min	0.36	0.25–0.44
Leukocytes max (10^9^/l)	10.4	3.9–31.2
CRP max (mg/l)	84.6	15.9–269.2
IL-6 max (pg/ml) (n = 29)	11.8	2.6–44.8
PTX3 max (ng/ml) (n = 42)	42.9	3.9–1085.5
IDO max (µmol/mmol) (n = 83)	202.2	47.7–3679.2
cf-DNA (µmol/ml) (n = 42)	1.35	1.04–3.29

Min = minimum, Max = maximum, suPAR = soluble urokinase-type plasminogen activator receptor, CRP = plasma C-reactive protein, IL-6 = plasma interleukin-6, PTX3 =  plasma pentraxin-3, IDO = serum indoleamin 2,3-dioxygenase, cf-DNA = plasma cell-free DNA.

**Figure 1:**
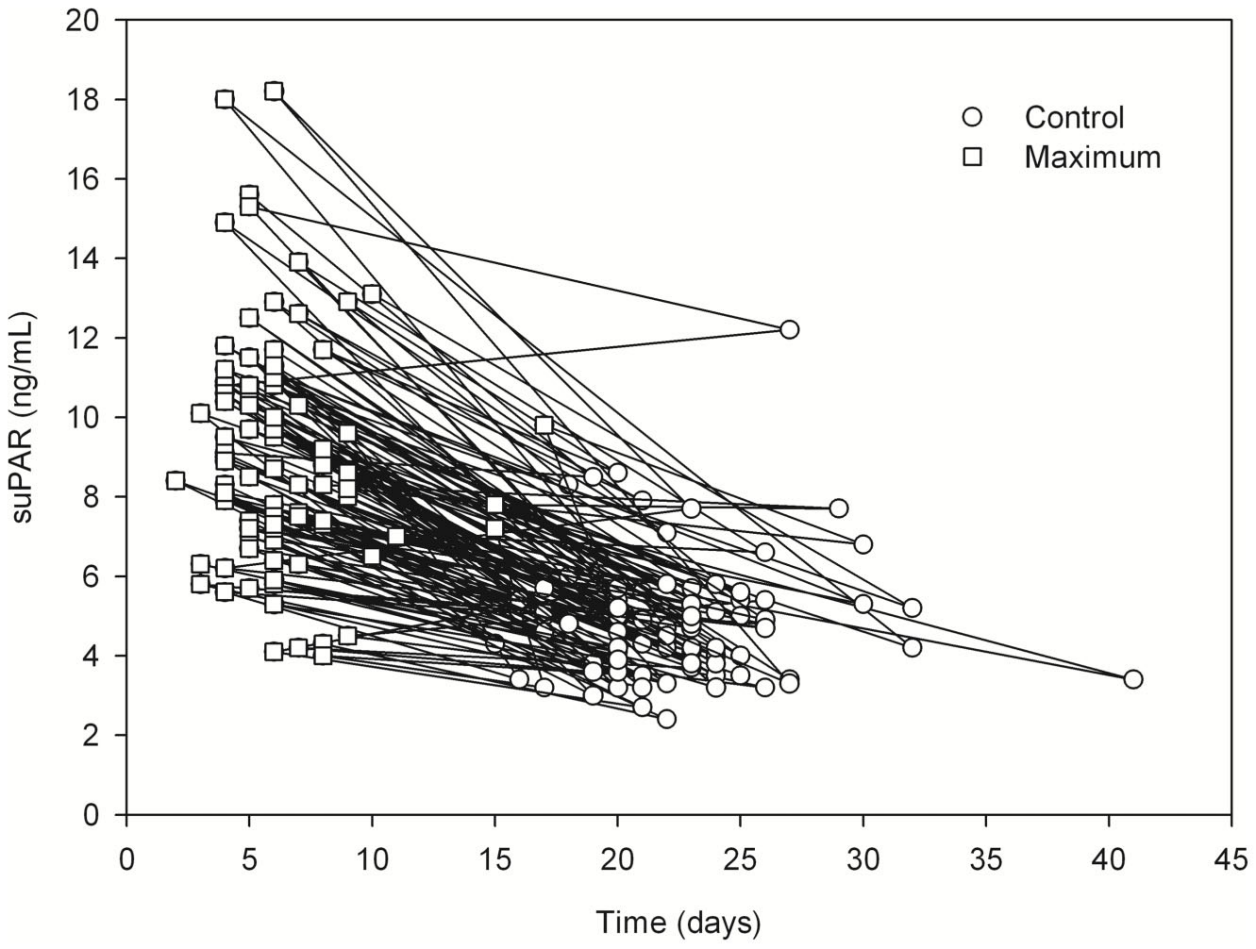
Line chart showing soluble urokinase-type plasminogen activator receptor (suPAR) maximum (median 8.7 ng/ml, range 4.0–18.2 ng/ml) and convalesce phase concentrations (median 4.7 ng/ml, range 2.4–12.2 ng/ml) in relation to the onset of fever (day 0) in 97 patients with Puumala hantavirus infection (P-value for the difference <0.001). Short title: Line chart showing suPAR maximum and convalescence concentrations in relation to the onset of fever.

The maximum plasma suPAR levels taken during acute NE were significantly elevated compared to the control values taken after the hospitalization period (median 8.7 ng/ml, range 4.0–18.2 ng/ml *vs.* median 4.7 ng/ml, range 2.4–12.2 ng/ml, *P*<0.001). The control values were taken median 22 (range 15–41) days after the onset of fever. Figure 1 shows the suPAR levels in relation to the duration of the disease, i.e. the onset of fever.

The maximum plasma suPAR levels correlated with several parameters reflecting the severity of acute PUUV infection (Table 3). There was a positive correlation of suPAR with the length of hospital stay and with the change in weight during hospitalization, which reflects fluid retention during the oliguric phase. Plasma suPAR also correlated positively with plasma creatinine, CRP, PTX3, IL-6, IDO, and cf-DNA levels, as well as with the blood leukocyte counts. An inverse correlation was detected between maximum plasma suPAR level and minimum urinary output as well as between maximum suPAR and minimum platelet levels. There was no correlation between maximum suPAR and age (Table 3).

**Table 3:** The correlations of maximum plasma suPAR levels with clinical and laboratory variables reflecting the severity of the infection in 97 patients with acute Puumala hantavirus infection.

	R	P-value
Length of hospitalization	0.325	0.001
Change in weight during hospitalization	0.406	<0.001
Urinary output min	−0.332	0.002
Creatinine max	0.378	<0.001
Platelets min	−0.325	0.001
Hematocrit min	−0.369	<0.001
Leukocytes max	0.475	<0.001
CRP max	0.298	0.003
PTX3 max	0.425	0.005
IL-6 max	0.621	<0.001
IDO max	0.557	<0.001
cf-DNA max	0.363	0.018

Min = minimum, Max = maximum, CRP = C-reactive protein, PTX3 = pentraxin-3, IL-6 = interleukin-6, IDO = indoleamine 2,3-dioxygenase, cf-DNA = cell-free DNA.

The maximum suPAR level was higher in patients who required dialysis treatment during hospitalization compared to patients who managed without dialysis (Table 4). Patients who stayed longest (>6 days) at the hospital had also higher suPAR levels than patients with shorter hospitalizations. Furthermore, patients with maximum creatinine level >200 µmol/l or maximum blood leukocyte count >10×10^9^/l had higher suPAR levels compared to patients with lower creatinine or leukocyte values. Significant thrombocytopenia (platelet count <50×10^9^/l) associated also with higher suPAR levels. There was no difference in suPAR levels between men and women (Table 4).

**Table 4:** Maximum plasma suPAR levels in different patient groups in 97 patients with Puumala virus infection.

	suPAR levels		
	Median	Range	P-value
**Sex**			0.841
Male	8.6	4.2–18.2	
Female	8.8	4.0–15.3	
**Dialysis**			0.038
Yes	10.8	8.6–18.0	
No	8.4	4.0–18.2	
**Hospital stay**			0.007
>6 days	9.2	6.5–18.2	
≤6 days	8.3	4.0–13.1	
**Minimum platelet level**			0.019
<50×10^9^/l	9.7	5.7–18.2	
≥50×10^9^/l	8.2	4.0–15.6	
**Maximum leukocyte count**			<0.001
>10×^9^/l	9.2	5.7–18.2	
≤10×^9^/l	7.5	4.0–13.1	
**Maximum creatinine**			0.001
>200 µmol/l	9.4	6.5–18.2	
≤200 µmol/l	8.0	4.0–15.6	

## Discussion

In the present study, plasma suPAR levels in a hantavirus infection are reported to our knowledge for the first time. The data presented here show that plasma suPAR levels are clearly elevated during acute PUUV infection. The suPAR values measured during the acute phase were markedly higher than the control values measured after the hospitalization and correspond with the levels previously reported in bacteremic patients [25]. Furthermore, the convalescence phase levels correspond to the levels previously measured in the general population [39]. The maximum plasma suPAR levels also correlated with several PUUV infection severity reflecting variables in the present study. suPAR correlated positively with the length of hospital stay, the change in body weight during hospitalization, plasma creatinine, and the leukocyte counts. An inverse correlation was detected by suPAR with minimum urinary output and minimum platelet levels.

There was also a positive correlation between suPAR and the biomarkers previously shown to predict the outcome of PUUV infection, PTX3, IL-6, IDO, and cf-DNA [16]–[19]. PTX3, IL-6, and IDO not only reflect the severity of PUUV induced NE, but also the activation of the immune system, and are partly expressed by the same type of cells as suPAR in response to inflammatory signals. Furthermore, cf-DNA reflects the degree of cellular damage. CRP, on the other hand, does not predict the outcome of PUUV infection [19]. In the present study, however, suPAR correlated also with CRP in PUUV-infected patients. This probably is explained by CRP reflecting the activation of the immune system, although it does not reflect the overall severity of the disease in PUUV infection.

Systemic suPAR levels are considered to reflect the degree of immunoactivation of the individual. This is corroborated by numerous studies showing suPAR levels to be increased as well in cancer as in various inflammatory and infectious diseases [22]. Furthermore, high suPAR concentrations are predictive of outcome and mortality in these conditions [22]. Previous studies on patients with infectious diseases have demonstrated that suPAR can predict disease severity and case fatality in malaria, tuberculosis, sepsis, bacterial meningitis, and some viral infections [24]–[27], [29], [30], [40]–[44].

Previously, suPAR has been studied only in a few viral infections. However, in HIV infection, several studies show that suPAR is a strong predictor of immunologic failure and mortality [28], [41]. In addition, the suPAR levels decrease with effective antiretroviral therapy [40]. In patients with Crimean-Congo hemorrhagic fever, suPAR is elevated and also predictive for mortality [42]. Systemic suPAR levels also predict the progression of liver fibrosis to cirrhosis in patients with chronic hepatitis C [45], [46]. Furthermore, elevated suPAR levels have been found in the cerebrospinal fluid of patients with viral meningitis [44]. Here, we demonstrate that suPAR is elevated and associates with severity of the disease in PUUV-induced hantavirus infection.

The exact pathogenetic mechanisms in hantavirus infection are currently still unclear. Pathological changes are marked by increased capillary permeability in the affected organs, which also explains many signs and symptoms in these infections [11]–[13]. The endothelium of the small vessels in various organs is the primary target in hantavirus infection, and the cellular entry of pathogenic hantaviruses is mediated by β_3_-integrins [11], [13]–[15]. Integrins are heterodimeric surface receptors on endothelial cells and platelets, mediating cell-to-cell adhesion, cell migration, extracellular matrix protein recognition, and platelet aggregation. β_3_-integrins have an important role in regulating vascular integrity, endothelial cell permeability, and platelet functions [47]. Pathogenic hantaviruses probably interfere with these functions, hence increasing endothelial permeability [47]. uPAR, in turn, interacts with integrins, including β_3_–integrins, and is believed to regulate their activation degree by altering their adhesive properties and signaling capacity [21], [22].

In this study, we found that plasma suPAR is markedly elevated in patients with PUUV-infection. Moreover, the higher the suPAR level is, the severer is also the disease. Taken into account the role of β_3_-integrins in the course of hantavirus infection, and on the other hand, the interactions of (s)uPAR and integrins, our finding presents as interesting. It brings up the possibility that increased suPAR is involved in the pathogenesis of PUUV infection by activating β_3_-integrins.

## Conclusions

The plasma level of suPAR is elevated in acute PUUV infection and associates with the severity of the disease. Therefore, plasma suPAR determinations may offer a potential diagnostic tool for assessing the severity and outcome of the disease. In addition, it is possible that circulating suPAR is involved in the pathogenesis of PUUV-induced hantavirus infection by activating β_3_-integrins. However, future studies are warranted to verify this assumption.
